# Retinale Zentralvenenthrombose bei einem jungen 21-jährigen Mann – Was steckt dahinter?

**DOI:** 10.1007/s00347-022-01703-6

**Published:** 2022-08-10

**Authors:** P. Werkl, M. Sommer, C. Singer, H. Tomasic, G. Seidel, N. Woltsche

**Affiliations:** Universitätsaugenklinik Graz, Medizinische Universität Graz, Auenbruggerplatz 4, 8036 Graz, Österreich

## Anamnese

Im November 2020 stellte sich ein 21-jähriger Mann in der Notaufnahme der Universitätsaugenklinik Graz vor. Als Beschwerden wurden Kopfschmerzen sowie ein plötzlich aufgetretener grauer Fleck im zentralen Gesichtsfeld des linken Auges geschildert, welcher erst seit wenigen Stunden bemerkt worden sei. Im Rahmen der Anamnese waren keinerlei ophthalmologischen Vorerkrankungen oder Traumata eruierbar. Bei der allgemeinen Anamnese wurden keine Vorerkrankungen oder eingenommenen Dauermedikamente angegeben – mit Ausnahme einer mild verlaufenen Infektion mit SARS-CoV‑2 (Severe acute respiratory syndrome coronavirus type 2) 2 Wochen zuvor. Es wurden ansonsten keine COVID-19(Coronavirus disease 2019)-spezifischen Beschwerden zum Zeitpunkt der Vorstellung an der Augenklinik angegeben.

## Befund

**Figure 1:**
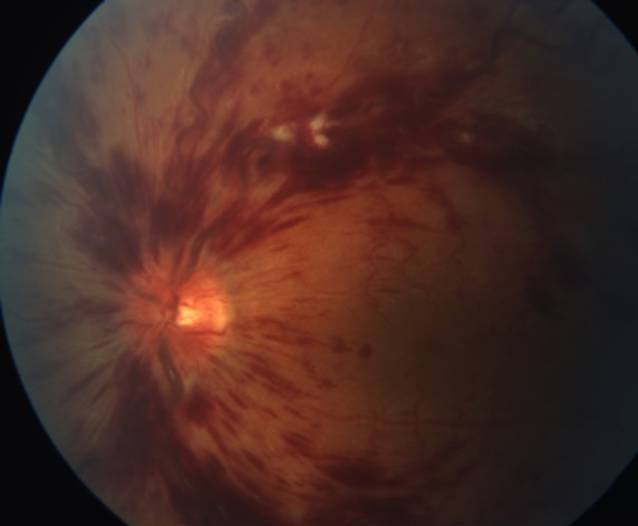

**Figure 2:**
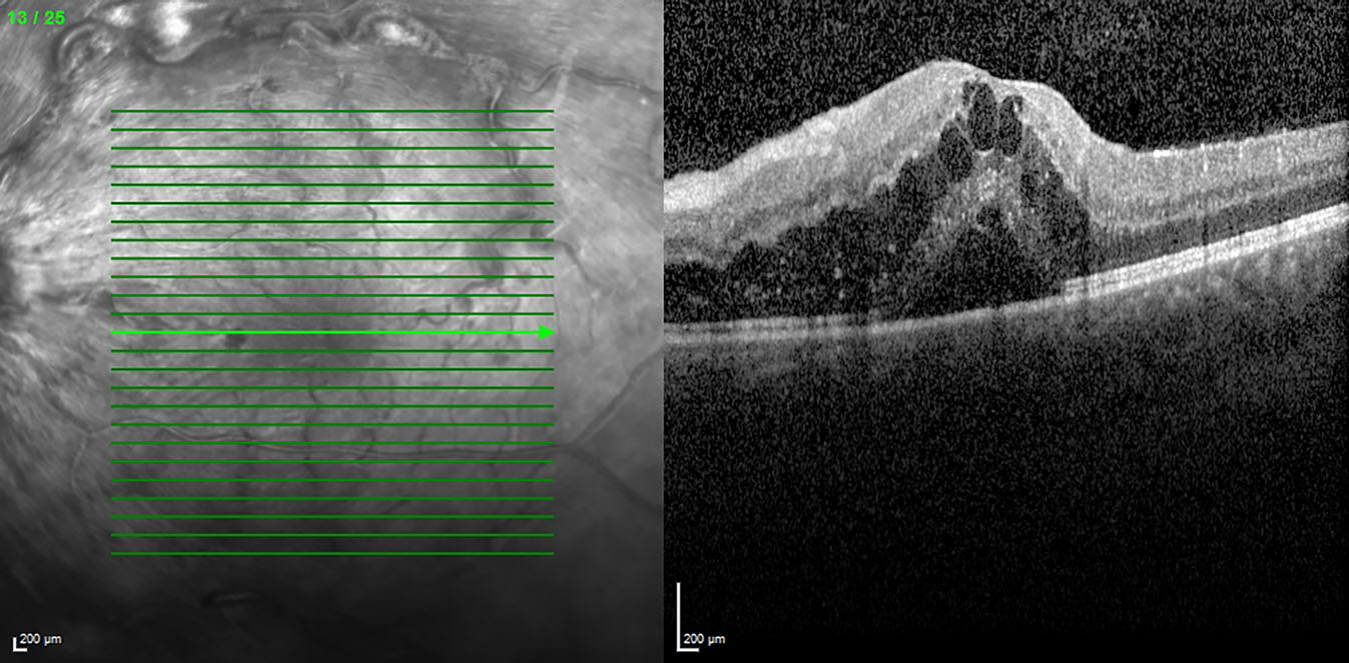

**Figure 3:**
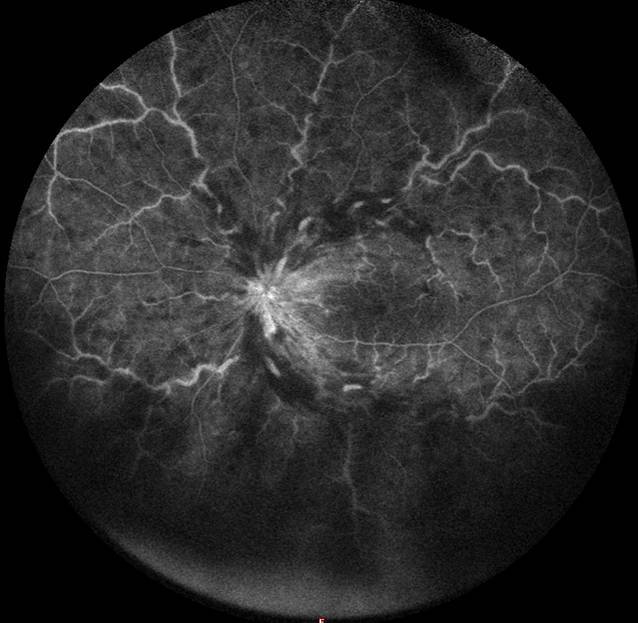

Im Rahmen der Untersuchung betrug die korrigierte Sehschärfe 20/50 (logMAR 0,4) am linken sowie 20/20 (logMAR 0) am rechten Auge. Spaltlampenmikroskopisch zeigte sich ein unauffälliger reizfreier vorderer Augenabschnitt beidseits, der Augendruck beidseits im Normbereich. Die Fundusuntersuchung in Mydriasis zeigte beidseits keine zelluläre Infiltration des Glaskörpers, jedoch links ausgeprägte flammenförmige intraretinale Blutungen entlang der Gefäßbögen und in allen Quadranten sowie eine Tortuositas der retinalen Venen in allen Quadranten, einzelne Cotton-wool-Spots und eine hyperäme Papille (Abb. 1). In der optischen Kohärenztomographie (OCT, Heidelberg Spectralis OCT®, Heidelberg Engineering GmbH, Deutschland) zeigte sich ein ausgeprägtes zystoides Makulaödem (Abb. 2) am linken Auge. In der durchgeführten Fluoreszeinangiographie zeigten sich tortuöse venöse retinale Gefäße mit dezenten perivaskulären Leckagen sowie eine Leckage im Bereich der Papille und zentral in der Makula. Die Hypofluoreszenzen in allen Quadranten waren vereinbar mit Blockaden der Fluoreszenz durch intraretinale Blutungen (Abb. 3). In der Fundoskopie sowie im OCT und in der Fluoreszeinangiographie des rechten Auges zeigten sich keine Auffälligkeiten und unauffällige retinale Gefäße.

Es wurden umgehend umfassende Laboruntersuchungen durchgeführt. Diese beinhalteten ein Blutbild sowie C‑reaktives Protein, Blutsenkung, Leber- und Nierenparameter, Elektrolyte, Fettstoffwechselparameter und ein Gerinnungslabor. Ein Screening bezüglich Thrombophilien erfolgte, darunter APC-Resistenz, Protein-S-Mangel und Protein-C-Mangel. D‑Dimer und Lupus-sensitive aktivierte partielle Thromboplastinzeit (Lupus aPTT) sowie Lupus-Antikoagulans (Lupus LA1) wurden analysiert. Auch ein Screening auf autoimmunologische Erkrankungen inklusive Bestimmung der antinukleären Antikörper (ANA), zytoplasmatischen Antikörper, ENA(„extractable nuclear antigen“)-Screening, ANCA(antineutrophile zytoplasmatische Antikörper)-Screening, sIL-2(„soluble“ Interleukin-2)-Rezeptor, Cardiolipinantikörper sowie S2-Glykoprotein-Screening, Untersuchung der Serumproteine, eine Serumeiweißelektrophorese und eine Untersuchung der Komplementfaktoren C3c sowie C4 wurden durchgeführt. Die Screeninguntersuchungen für Thrombophilie und Autoimmunerkrankungen zeigten keine Auffälligkeiten. Auch wurden infektiöse Erkrankungen wie eine virale Hepatitis, HIV, eine Borreliose oder Tuberkulose (mittels Quantiferon-Test) ausgeschlossen. Insgesamt zeigten sich die Laboruntersuchungen unauffällig bis auf einen erhöhten Triglyzeridwert von 269 mg/dl sowie einen gering erhöhten Cholesterinwert von 231 mg/dl (HDL- und LDL-Cholesterin waren im Normbereich). Im Rahmen der Familienanamnese konnte der Patient keine nähere Auskunft über internistische Erkrankungen der Angehörigen geben.

Eine umfassende Durchuntersuchung mit 24-h-Blutdruckmessung durch den betreuenden niedergelassenen Internisten zum Ausschluss möglicher anderweitiger Ursachen wurde veranlasst. Im Rahmen der internistischen Untersuchung zeigte sich eine milde arterielle Hypertonie, welche zur Einleitung einer milden antihypertensiven Monotherapie führte, ansonsten war auch diese unauffällig, und es war keine weitere therapeutische Intervention oder Abklärung von internistischer Seite aus erforderlich. Die von uns empfohlene 24-h-Blutdruckmessung erfolgte daher nicht.

## Diagnose

Zentralvenenthrombose mit Makula- und Papillenödem am linken Auge nach SARS-CoV-2-Infektion.

## Therapie und Verlauf

Bei vorliegendem Befund wurden dem Patienten 2 intravitreale Injektionen mit Bevacizumab (Avastin®, 1,25 mg/0,05 ml) im Abstand von 4 Wochen empfohlen. Aufgrund der Seltenheit der vorliegenden Entität, einer Zentralvenenthrombose nach einer rezenten SARS-CoV-2-Infektion, beschränkte sich auch die Literatur zum damaligen Zeitpunkt auf einzelne wenige Fallberichte. Ein Fallbericht von Sheth et al. berichtete über eine retinale Zentralvenenthrombose bei einem 52-jährigen Mann mit schwerer COVID-Erkrankung 3 Tage nach stationärer Entlassung, welcher mit systemischen Kortikosteroiden (40 mg pro Tag) in Kombination mit intravitrealem Anti-VEGF Razumab® behandelt wurde, was zu einer kompletten Remission des Makulaödems führte [1]. Nach Durchsicht der Literatur sowie eingehender Diskussion wurde der Entschluss gefasst, eine alleinige Therapie mit intravitrealem Anti-VEGF zu beginnen, da retinale Venenverschlüsse bekanntermaßen mit deutlich erhöhten intravitrealen VEGF-Spiegeln einhergehen, welche durch intravitreale Applikation von Anti-VEGF deutlich gesenkt werden können, und diese somit die etablierte First-line-Therapie bei retinalen Venenthrombosen mit Makulaödem darstellen [2].

**Figure 4:**
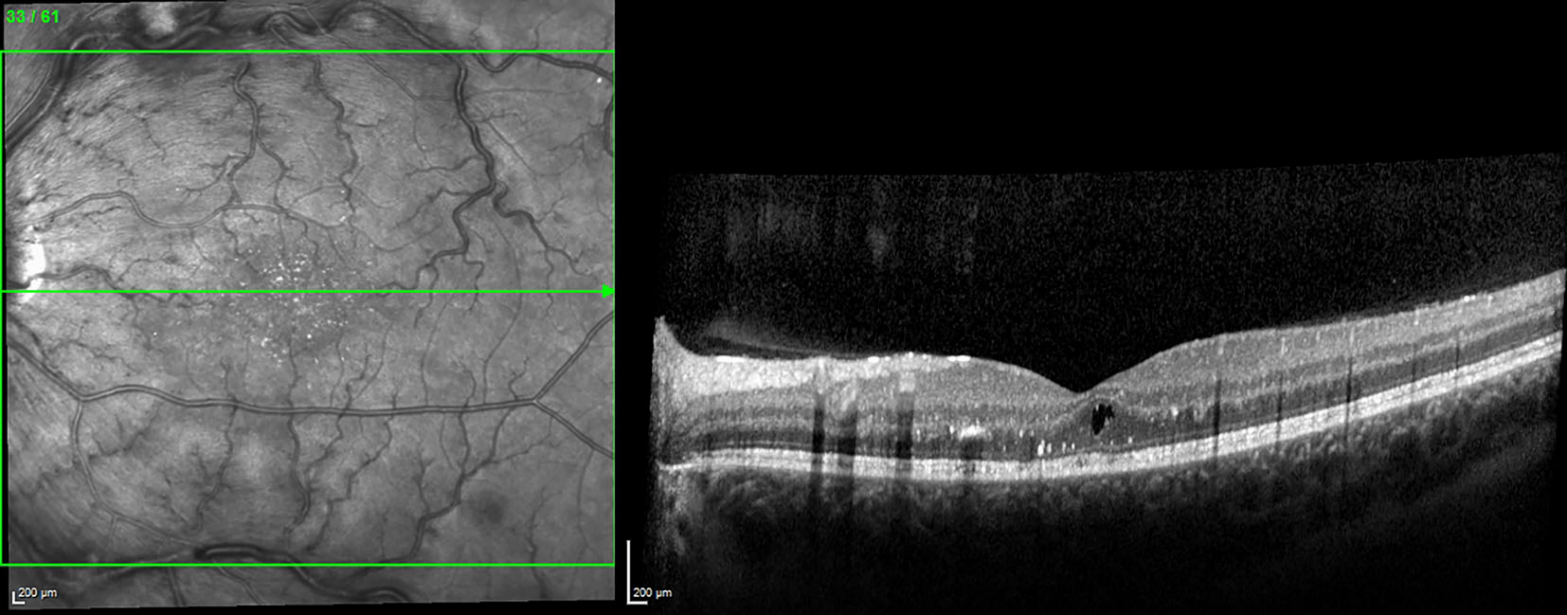

Im Follow-up 4 Wochen nach der zweiten Injektion zeigte sich ein kompletter Rückgang des Makulaödems bei einem Visus von 20/25 links (logMAR 0,1). Bei einem erneuten Kontrollbesuch im April 2021 zeigte sich ein stabiler Visus bei jedoch wiederum vereinzelten zentralen intraretinalen Zysten (Abb. 4). Daraufhin wurden 2 weitere Injektionen mit Bevacizumab empfohlen und durchgeführt. In einer späteren Kontrolle im Juli 2021 zeigte sich eine komplette Remission des Makulaödems bei einem Visus von 20/20 (logMAR 0).

Weitere monatliche augenärztliche Kontrollen wurden empfohlen mit der Möglichkeit einer Wiedervorstellung an der Universitätsaugenklinik bei Bedarf, welche jedoch bis zum Mai 2022 nicht in Anspruch genommen werden musste. Bei einer telefonischen Kontaktaufnahme mit dem Patienten im April 2022 gab er Beschwerdefreiheit an.

## Diskussion

Eine Zentralvenenthrombose im Alter von 21 Jahren ist ein ungewöhnlicher Befund. Es lagen beim Patienten zwar Risikofaktoren vor (milde arterielle Hypertonie, erhöhte Triglyzeridwerte), die jedoch allein angesichts des jungen Alters nicht ausreichend für die Verursachung einer Zentralvenenthrombose erscheinen. Jedoch ist die zeitliche Assoziation mit einer SARS-CoV-2-Infektion ein wichtiger zu berücksichtigender Faktor in der Diagnosestellung. SARS-CoV‑2 werden mehrere Pathomechanismen in der Entstehung von Thrombosen nachgesagt: Der primäre zelluläre Rezeptor für SARS-CoV‑2, der Angiotensin-Converting-Enzyme-2-Rezeptor (ACE-2-Rezeptor) ist auch in der humanen Retina zu finden. In retinalen Biopsien von COVID-19-Patient*innen konnte das Virus in einzelnen Fällen nachgewiesen werden [3]. Der für die Venenthrombose ursächliche Pathomechanismus könnte einerseits eine direkte Wirkung von viralen Partikeln im Rahmen einer Vaskulitis oder andererseits eine aktivierte thromboinflammatorische Kaskade im Rahmen eines „Cytokine-Storm“ der Immunantwort auf virale Partikel sein. Zweiteres könnte das verzögerte Auftreten von Thrombosen nach einer abgelaufenen Infektion erklären [4, 5].

Von einer in der Literatur beschriebenen Herangehensweise in einem ähnlichen Fall mit systemischen Steroiden kombiniert mit intravitrealem Anti-VEGF [1] wurde in unserem Fall abgesehen, da zum Zeitpunkt der Vorstellung unseres Patienten keinerlei sonstige systemische Beschwerden vorlagen. Aufgrund des möglichen Nebenwirkungsprofils sowie nicht vorhandener Evidenz aus größeren Studien entschieden wir uns für die alleinige intravitreale Anti-VEGF-Therapie und konnten damit ein zufriedenstellendes Outcome erzielen.

Nun ist durch die multifaktorielle Genese einer retinalen Venenthrombose in diesem Fall nicht gänzlich zu eruieren, ob die Thrombose alleine durch die SARS-CoV-2-Infektion entstanden ist. Jedoch besteht aufgrund der geschilderten Mechanismen, der zeitlichen Korrelation, des Patientenprofils und der derzeit noch begrenzten, jedoch vorliegenden Fallberichte von retinalen Venenthrombosen in Assoziation mit SARS-CoV‑2 der naheliegende Verdacht auf eine Kausalität. Wie in diesem Fall werden die übliche umfassende systemische und internistische Abklärung und Ursachenforschung zum Ausschluss anderer zugrunde liegender Erkrankungen und Risikofaktoren immer empfohlen.
